# Fast and high resolution single-cell BRET imaging

**DOI:** 10.1038/srep28231

**Published:** 2016-06-15

**Authors:** Elise Goyet, Nathalie Bouquier, Vincent Ollendorff, Julie Perroy

**Affiliations:** 1 CNRS, UMR-5203, Institut de Génomique Fonctionnelle, Montpellier, F-34094, France; 2 INSERM, U1191, Montpellier, F-34094, France; 3 Universités de Montpellier, UMR-5203, Montpellier, F-34094, France; 4 INRA, UMR866 Dynamique Musculaire et Mébabolisme, Université Montpellier, 34060 Montpellier, France

## Abstract

Resonance Energy Transfer (RET)-based technologies are used to report protein-protein interactions in living cells. Among them, Bioluminescence-initiated RET (BRET) provides excellent sensitivity but the low light intensity intrinsic to the bioluminescent process hampers its use for the localization of protein complexes at the sub-cellular level. Herein we have characterized the methodological conditions required to reliably perform single-cell BRET imaging using an extremely bright luciferase, Nanoluciferase (Nluc). With this, we achieved an unprecedented performance in the field of protein-protein interaction imaging in terms of temporal and spatial resolution, duration of signal stability, signal sensitivity and dynamic range. As proof-of-principle, an Nluc-containing BRET-based sensor of ERK activity enabled the detection of subtle, transient and localized variations in ERK activity in neuronal dendritic spines, induced by the activation of endogenous synaptic NMDA receptors. This development will improve our comprehension of both the spatio-temporal dynamics of protein-protein interactions and the activation patterns of specific signaling pathways.

The life of a cell is ruled by the dynamics of its molecular actors. While multiple external stimuli can share a narrow repertoire of signaling molecules, the fine-tuning of protein complex remodeling in terms of space and time can determine the specificity of cellular responses. Bioluminescence Resonance Energy Transfer (BRET) relies on the energy transfer from a bioluminescent enzyme, a luciferase, and a fluorophore. Unlike FRET (Fluorescence Resonance Energy Transfer), BRET is initiated by an enzymatic reaction and thus does not require light excitation, resulting in an excellent signal to background ratio and greater sensitivity^1^. This therefore provides a powerful tool for studying the temporal conformational changes and direct protein-protein interactions in the natural environment of living cells. Importantly, the precise spatial dynamics of a molecular signaling at the sub-cellular level is also fundamental to understanding the functional impact of specific molecular signaling pathways on the cell’s biological response. We previously adapted BRET technology for use with microscopy to precisely define the spatial dynamics of protein-protein interactions in real time at the subcellular level^2^,^3^. However, the major limitation of this technique has remained the poor temporal resolution, resulting from the difficulty in detecting the low number of photons emitted by the catalytic oxidation of the *Renilla* luciferase (Rluc) substrate. Consequently, despite a high sensitivity, BRET imaging can currently only be used to investigate stable interactions (in the minutes range).

Numerous efforts have been made to develop improved luciferase enzymes that are better suited to bioluminescence imaging^4^. Among them, Rluc8, a mutant variant of Rluc with improved stability and brightness, is currently one of the most commonly used luciferases in BRET assays^5^. More interestingly, the development of Nanoluciferase (Nluc), which produces an intense and sustained luminescence signal, allows the accurate quantification of very small numbers of entities^6^,^7^. The physical properties of Nluc, especially its stability and luminescence efficiency, have dampen the field of bioluminescence these last months in a wide array of applications such as detection of gene expression, protein stability and protein-ligand interaction (for review^8^). The brightness of Nluc also opens up new possibilities for bioluminescence imaging. For example, it has been used to track viral infection^9^ or tumor growth^10^ and monitor disease progression. Finally, Nluc gave birth to a generation of sensitive biosensors either using split Nluc to report proteins-protein interaction^11^, or using BRET to monitor conformational changes during ion channel activation in real time^12^. Nluc promising features should exceed the best BRET^13^- and FRET^14^-based sensors so far described.

In the present work, we characterized the methodological conditions required to reliably report single-cell BRET imaging using Rluc8 or Nluc donors genetically fused to the Venus acceptor. The use of Nluc greatly improved the resolution in terms of both time and space, enhanced the duration of signal stability, expanded the dynamic range and increased the sensitivity of the BRET signals in single-cell imaging. To illustrate the benefits of using Nluc in BRET imaging, we designed an Nluc-optimized BRET-based sensor of ERK activity. This reporter displayed a higher sensitivity and improved spatio-temporal resolution, enabling the detection of subtle, transient and localized variations in ERK activity in the dendritic spines of hippocampal neurons.

## Results

### Optimized BRET imaging with Nluc

**Figure 1:**
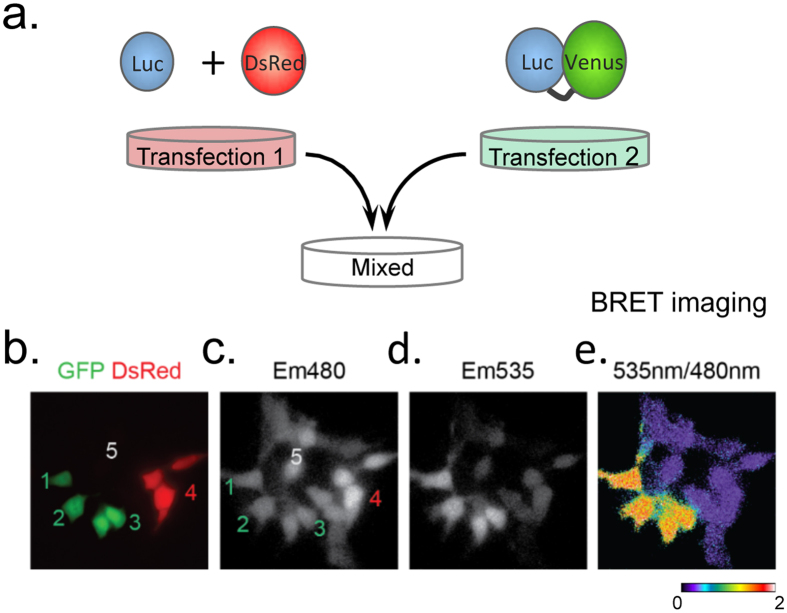
Experimental protocol scheme. **(a)** Cells were transfected with the donor entity and a red-fluorescent transfection reporter, DsRed (left) or the BRET-positive fusion (right). These two populations of transfected cells were subsequently mixed (24 hours after transfection). The following illustrations **(b–e)** were obtained with Nluc as BRET donor. **(b)** Cells expressing the donor, Nluc (+DsRed), or the donor fused to the acceptor, Nluc-Venus, could be identified by direct fluorescence excitation (GFP and DsRed merged image) when studied in the same microscopic field, allowing a direct comparison of cells that did or did not display BRET. In the absence of light excitation, Em480 **(c)** and Em535 **(d)** images were acquired in the presence of the relevant luciferase substrate. **(e)** The pixel-by-pixel ratio of images obtained at 535 nm, compared to those obtained at 480 nm, demonstrates a strong signal in cells expressing the BRET-positive fusion (cells 1, 2 and 3), but only a marginal signal in cells expressing the donor alone (cells 4 and 5). As a control it must be noted that some cells from the DsRed + luciferase-transfected population which expressed only luciferase displayed the same 535 nm/480 nm ratio as cells co-expressing DsRed with luciferase.

First, the Nluc spectrum was analyzed and found to display promising features that would allow a BRET signal to be monitored at the single-cell level (Fig. S1). We therefore explored the conditions required to accurately image the BRET signal with Nluc as the donor. To distinguish the transfer of energy signal from the signal resulting from an overflow of the energy donor output into the energy acceptor detection channel, we measured the Em535/Em480 ratios from the same microscopic field of cells expressing either the BRET donor alone (Rluc8 or Nluc co-transfected with DsRed as a transfection reporter, basal ratio) or expressing the donor genetically fused to the Venus acceptor (Rluc8-Venus or Nluc-Venus, BRET-positive fusions) (Fig. 1). We then compared the temporal resolution, kinetics and sensitivity of single-cell BRET imaging in cells expressing Nluc-Venus versus Rluc8-Venus.

#### Temporal resolution

**Figure 2:**
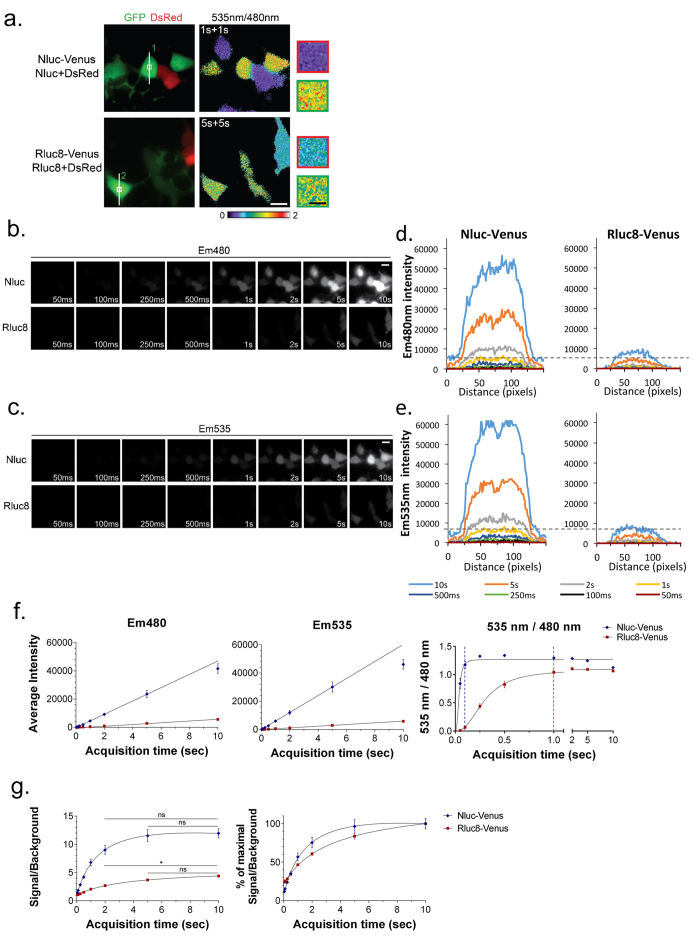
The time resolution of BRET imaging is 10-times greater with Nluc compared to Rluc8. **(a)** HEK cells were co-transfected with either the BRET fusions Nluc-Venus (top) or Rluc8-Venus (bottom), or the donor entities Nluc (top) or Rluc8 (bottom), together with the DsRed transfection reporter. On the left, GFP and DsRed fluorescence (merged images) was used to discriminate between cells expressing the BRET fusion with those expressing the donor entity with DsRed. On the right, the 535 nm/480 nm ratio image was obtained from either a 1 s or 5 s sequential acquisition per channel, 5 min after the addition of the relevant substrate, Furimazine (top) or Coelenterazine H (bottom). Square areas are shown at a higher magnification in the right panels. Scale bars: white = 20 μm; black = 5 μm. **(b)** Em480 and **(c)** Em535 images obtained for acquisition times ranging from 50 ms to 10 s were recorded 5 min after the addition of Coelenterazine H or Furimazine. **(d)** Em480 and **(e)** Em535 intensities recorded for the indicated acquisition times along a cross-section of cells 1 (left) and 2 (right) in A. **(f)** Average Em480, Em535, and 535 nm/480 nm signal intensities, expressed as a function of acquisition time per channel for Nluc-Venus or Rluc8-Venus. Acquisition times over 1 s per channel were required to detect reliable BRET signals with Rluc8 while an acquisition time of 100 ms was sufficient with Nluc. **(g)** Em480 signal/background ratio absolute values (left), or normalized to their maximal value (right). Nluc produced a higher signal/background ratio than Rluc8. A 1.7 s acquisition time was required to reach 50% of the signal/background ratio with Nluc-Venus versus 3.2 s for Rluc8-Venus. **(f, g)** Each point of the graph represents the mean ± SEM obtained from 5 to 7 cells of similar fusion expression levels (YFP intensities: Nluc-Venus = 745 ± 28 RLUs; Rluc8-Venus = 771 ± 33 RLUs), from 3 square areas per cell.

Currently, the major limitation of single-cell BRET imaging is the weak donor luminescence which leads to prolonged acquisition times. Therefore, we first assessed the benefits of Nluc compared to Rluc8 on BRET temporal resolution. To determine the minimal acquisition time required to detect the BRET signal, we performed sequential acquisitions of donor (Em480) and acceptor (Em535) emissions with acquisition times ranging from 50 ms to 10 s (Fig. 2). These recordings started 5 min after incubation with the relevant luciferase substrates since at this time the luminescence signal was at its maximum intensity and the BRET signals were constant (Fig. S2a–c). As expected, the Em480 and Em535 signals increased linearly with acquisition time for both the Nluc and Rluc8 luciferases (Fig. 2f). To obtain the Em480 and Em535 signals, we measured the intensity of fluorescence along lines drawn across cells expressing Nluc-Venus and Rluc8-Venus with similar Venus-fluorescence intensities, assuming that similar intensities reflected similar expression levels of fusion protein. A typical field of view is shown in Fig. 2. In this figure, the Venus fluorescence of Nluc-Venus (cell 1) was 672.12 ± 0.55, compared to 897.88 ± 1.29 for Rluc8-Venus (cell 2, Fig. 2a). Despite similar expression levels, the Em480 intensity collected from a 1 s acquisition with Nluc (4769 ± 32 RLU) was similar to that obtained in 5 s (3924 ± 35 RLU) or 10 s (8048 ± 41 RLU) with Rluc8 (Fig. 2d). This example is representative of the average intensities measured on several cells expressing similar amounts of fusion proteins (Fig. 2f). Em480 and Em535 signals were found to be in the linear detection range of the camera for acquisition times ranging from 100 ms to 5 s with Nluc-Venus, but only those longer than 1 s for Rluc8-Venus. Accordingly, pixel by pixel division of Em535 and Em480 signals showed that the constant 535 nm/480 nm ratios of Nluc-Venus were efficiently detected with acquisition times as short as 100 ms per channel while acquisition times longer than 1 s per channel were required for Rluc8-Venus (Fig. 2f). For each acquisition time, the signal/background values of Em480 were higher with Nluc than Rluc8 (Fig. 2g, left panel). These values were not significantly improved for acquisition times above 2 s for Nluc and 5 s for Rluc8, indicating that longer acquisition times would not enhance the quality of images (Fig. 2g, right panel). Taken together, these results show that the time resolution of BRET imaging is 10-times greater with Nluc as the bioluminescent energy donor. The use of Nluc should therefore allow the study of fast, transient interactions between proteins.

#### Kinetics of the BRET signal

**Figure 3:**
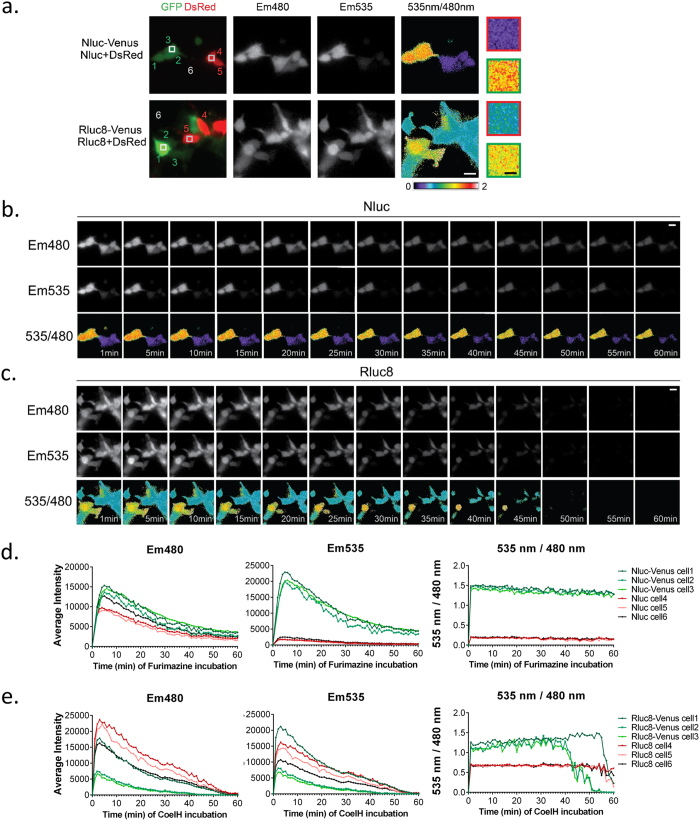
Nluc generates a stable BRET signal over time in single-cell imaging. (**a)** Merged images of YFP and DsRed fluorescence were used to discriminate between cells expressing the BRET fusions Nluc-Venus (top) or Rluc8-Venus (bottom) (cells 1, 2 and 3), and cells expressing the donor + DsRed (cells 4 and 5) or the donor alone (cell 6). Em480, Em535 and the 535 nm/480 nm pseudo-colored ratio images were recorded with 1 s (top) or 5 s sequential acquisitions per channel (bottom) after 5 min incubation with the relevant substrate. Square areas are shown at a higher magnification in the right panels. **(b,c)** Em480, Em535 and the 535 nm/480 nm ratio images were acquired every 5 min from 0 to 60 min after substrate incubation with cells expressing Nluc and Nluc-Venus **(b)** or Rluc8 and Rluc8-Venus **(c**). **(d,e)** The intensity of Em480, Em535, and 535 nm/480 nm ratio signals were measured every minute for 60 min with Nluc **(d)** and Rluc8 **(e)** as the energy donors within a square area of cells numbered in (**a**). White scale bar = 20 μm; Black scale bar = 5 μm.

To characterize the performance of Nluc in the evolution of the BRET signal over time, we performed repeated sequential acquisitions of Em480 and Em535 every minute for 60 min with 1 s and 5 s acquisition times per channel for Nluc and Rluc8 respectively (Fig. 3). The light intensity varied from one cell to another, depending on the protein expression level. For both Rluc8- (Fig. 3b,d) and Nluc- (Fig. 3c,e) expressing cells, Em480 and Em535 reached a maximum within 4 min of luciferase substrate incubation, then slowly decreased. Importantly, Em480 and Em535 signals originating from the oxidation of Coelenterazine H by Rluc8 (Fig. 3e) decreased faster than those originating from the oxidation of Furimazine by Nluc (Fig. 3d). Rluc8 emission reached the lower detection limits of the camera in 40 to 45 min while Nluc emission remained within the linear detection range for more than 1 hour of recording (up to 3 hours, data not shown). Consequently, the constant 535 nm/480 nm ratios could be accurately measured for 40 min of recording with Rluc8 and more than 60 min with Nluc (Fig. 3d,e). It is important to note that for any given transfection conditions, the 535 nm/480 nm ratio intensity did not depend on protein expression levels and was constant in all cells recorded (Fig. 3d,e). Thus, since the luminescence signal resulting from the oxidation of Furimazine by Nluc decreased slowly, Nluc therefore provided a better stability of BRET signal over time compared to Rluc8. These results show that Nluc will enable longer duration studies of protein-protein interaction dynamics.

#### Gain in BRET sensitivity

**Figure 4:**
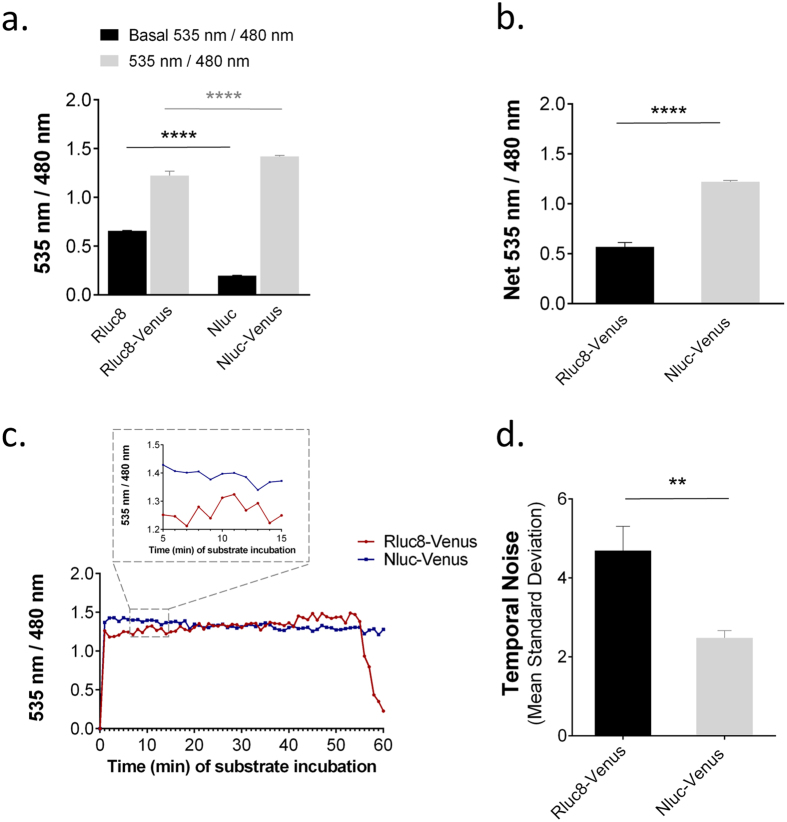
Nluc improves the sensitivity and dynamic range of BRET measurements in single-cell imaging. **(a)** Bar graph of the average 535 nm/480 nm ratio intensities of Rluc8, Rluc8-Venus, Nluc and Nluc-Venus obtained from 5 s (Rluc8) or 1 s (Nluc) sequential acquisitions per channel between 5 and 20 min after substrate addition. Nluc decreased the basal 535 nm/480 nm ratio. Nluc triggered a more efficient energy transfer with Venus compared to Rluc8. **(b)** Average of the net 535 nm/480 nm ratio intensities for Rluc8-Venus and Nluc-Venus calculated by subtracting the basal 535 nm/480 nm ratio obtained with Rluc8 and Nluc from the 535 nm/480 nm ratio of Rluc8-Venus and Nluc-Venus respectively. The dynamic window for 535 nm/480 nm ratio measurements was significantly better using Nluc compared to Rluc8. **(c)** Average 535 nm/480 nm ratio intensity measured every minute for 60 min after substrate addition in the square area shown in cells expressing Rluc8-Venus (cell 1) and Nluc-Venus (cell 3) in Fig. 3a. Close-up of the 5 to 15 min time period after substrate addition when the 535 nm/480 nm ratio was stable. **(d)** Mean standard deviation of the 535 nm/480 nm ratios between 5 and 20 min after substrate incubation, expressed as a percentage of the mean ratio signal. The temporal noise was significantly lower with Nluc than Rluc8. Bars represent mean ± SEM obtained from 8 or 9 cells. Mann & Whitney statistical analysis: **p < 0.01, ****p < 0.0001.

As highlighted by the emission spectra (Fig. S1), the greater spectral separation together with the higher efficiency of energy transfer within the Nluc-Venus fusion suggested an enlarged dynamic window. Cells expressing Nluc, Rluc8, Nluc-Venus or Rluc8-Venus were easily distinguishable by their specific 535 nm/480 nm ratios (Fig. 3d,e). Hence, we quantified the benefits of Nluc in terms of the sensitivity of BRET signals by comparing the energy transfer efficiency initiated by Rluc8 or Nluc during a time period when the BRET signals from both of these donors were stable (between 5 and 20 min of substrate incubation, Fig. 3d,e). First, the basal 535 nm/480 nm ratio in cells expressing the donor alone was found to be significantly smaller with Nluc compared to Rluc8 (0.1978 ± 0.0039 versus 0.6565 ± 0.0055 respectively, Fig. 4a). In addition, the 535 nm/480 nm ratio was significantly increased in cells expressing the Nluc-Venus fusion compared to cells expressing the Rluc8-Venus (1.42 ± 0.01 versus 1.22 ± 0.05, Fig. 4a). Therefore, the net 535 nm/480 nm ratio was remarkably higher for the Nluc-Venus fusion compared to the Rluc8-Venus fusion (1.21 ± 0.01 versus 0.56 ± 0.05), reflecting an enlarged dynamic window of BRET (Fig. 4b), as illustrated by the pseudo-colored representations in Figs 2a and 3a.

To quantify the noise, we measured over time the standard deviation of the 535 nm/480 nm ratio signal from one measure to the next, at a time period when the BRET signal was known to be stable (between 5 and 20 min incubation with the luciferase substrates, Fig. 4c). For cells containing similar amounts of BRET-positive constructs (with similar luminescence intensities, obtained with a 5 s and 1 s acquisition time for Rluc8-Venus and Nluc-Venus, respectively), Nluc had approximately half the amount of temporal noise (Fig. 4c,d).

Taken together, these results strongly favor the use of Nluc for BRET imaging. The greater luminescence intensity of Nluc and its left-shifted spectrum substantially improved both the time resolution and stability of the BRET signal over time, while the temporal noise was reduced and the dynamic window enlarged. These benefits should allow the detection of subtle and transient changes to protein-protein interactions that other luciferases have failed to report until now. Moreover, from the gain in temporal resolution we can also expect an improved spatial resolution of BRET images, given the fact that the mobility of proteins in living cells will be limited with shorter acquisition times.

### Improved sensing of ERK activity

**Figure 5:**
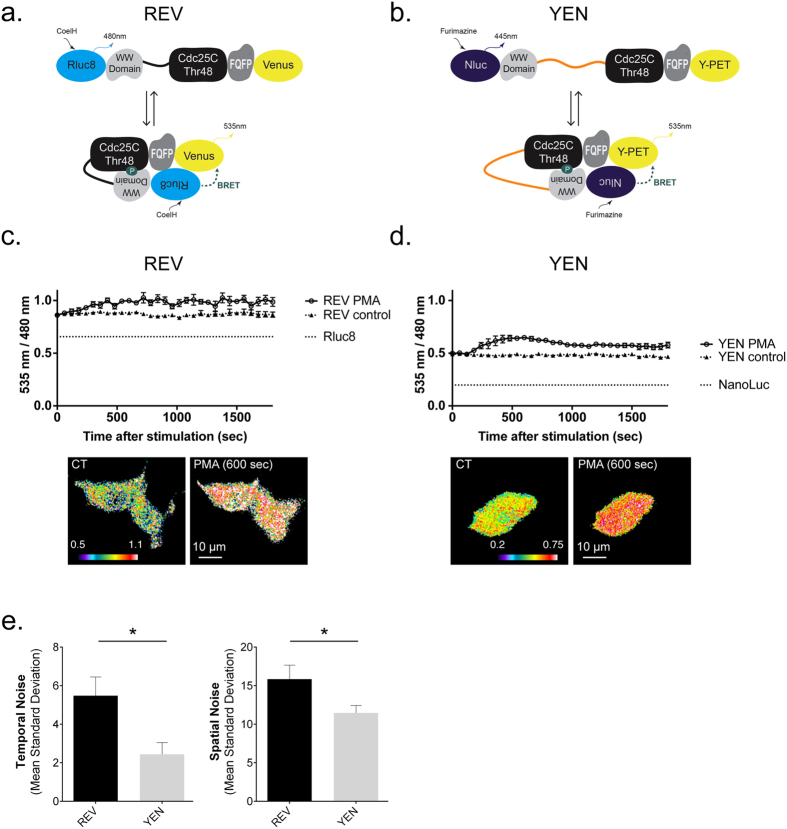
The Nluc-containing ERK-activity reporter improves the sensitivity and spatio-temporal resolution of BRET signals. **(a,b)** Schematic representations of the conformational changes of the REV **(a)** and YEN **(b)** sensors induced upon ERK activation. Adapted from Xu *et al*.^15^. **(c,d)** Average 535 nm/480 nm ratio intensity measured every minute for 30 min after PMA (PMA) or buffer (control) addition to REV- **(c)** and YEN- **(d)** transfected cells. 535 nm/480 nm ratio images obtained before (CT) and after 10 min PMA application (PMA (600 sec)). **(e)** Mean standard deviation of the 535 nm/480 nm ratios in time (left) and space (right), expressed as a percentage of the mean ratio signal. Left- The temporal noise was measured from 1200 to 1620 s after PMA incubation. Right- The spatial noise was measured 600 s after PMA application within a square area of 21 × 21 pixels. Bars are mean ± SEM obtained from 6 cells. Mann & Whitney statistical analysis: *p < 0.05.

To validate the observed performance of Nluc-initiated BRET in monitoring sub-cellular protein-protein interactions, we engineered a novel BRET-based sensor of ERK activity containing Nluc, which we called YEN (see methods). Briefly, in its unphosphorylated form the reporter adopts an “open” conformation. Upon phosphorylation, conformational bending to adopt the “closed” conformation increases the proximity between the donor and acceptor and induces an increase in BRET to report its phosphorylation by ERK (Fig. 5a,b). The efficiency of the YEN sensor in reporting ERK activity was first compared to the Rluc8 version, REV^15^, in HEK cells. Under basal conditions, both reporters displayed a significantly higher 535 nm/480 nm ratio (0.4898 ± 0.0048 for YEN and 0.8726 ± 0.0026 for REV, Fig. 5c,d) than the basal 535 nm/480 nm ratio recorded in cells expressing luciferases alone (0.1978 ± 0.0039 for Nluc and 0.6565 ± 0.0055 for Rluc8, Fig. 4a). It has previously been shown that this intra-molecular BRET at resting state reports both proximity between donor and acceptor entities of the unphosphorylated-open conformation of the reporter, but also a low phosphorylation of REV and YEN due to a basal activity of ERK^15^.

Phorbol myristate acetate (PMA)-induced ERK activation significantly increased the 535 nm/480 nm ratio in both REV- and YEN-transfected cells (maximal PMA-induced 535 nm/480 nm ratio increase was 0.1736 ± 0.0042 for YEN, 0.1679 ± 0.0061 for REV, Fig. 5c,d). Interestingly, the standard deviation of the 535 nm/480 nm ratio (temporal noise) from one measure to the next when the BRET signal was stable was reduced 2-fold with YEN (2.44 ± 0.61% of the signal) compared to REV (5.48 ± 0.97% of the signal, Fig. 5e, left panel), giving rise to a better-defined kinetic evaluation of ERK activation following PMA stimulation over time. Assuming a homogenous activity of ERK in HEK cells, the spatial noise among pixels at a given time was also significantly lower with YEN (11.47 ± 0.97% of the signal, 600 s after PMA application) compared to REV (15.86 ± 1.81% of the signal, Fig. 5e, right panel). These results emphasize the improved sensitivity and spatio-temporal resolution of BRET signals with YEN.

**Figure 6:**
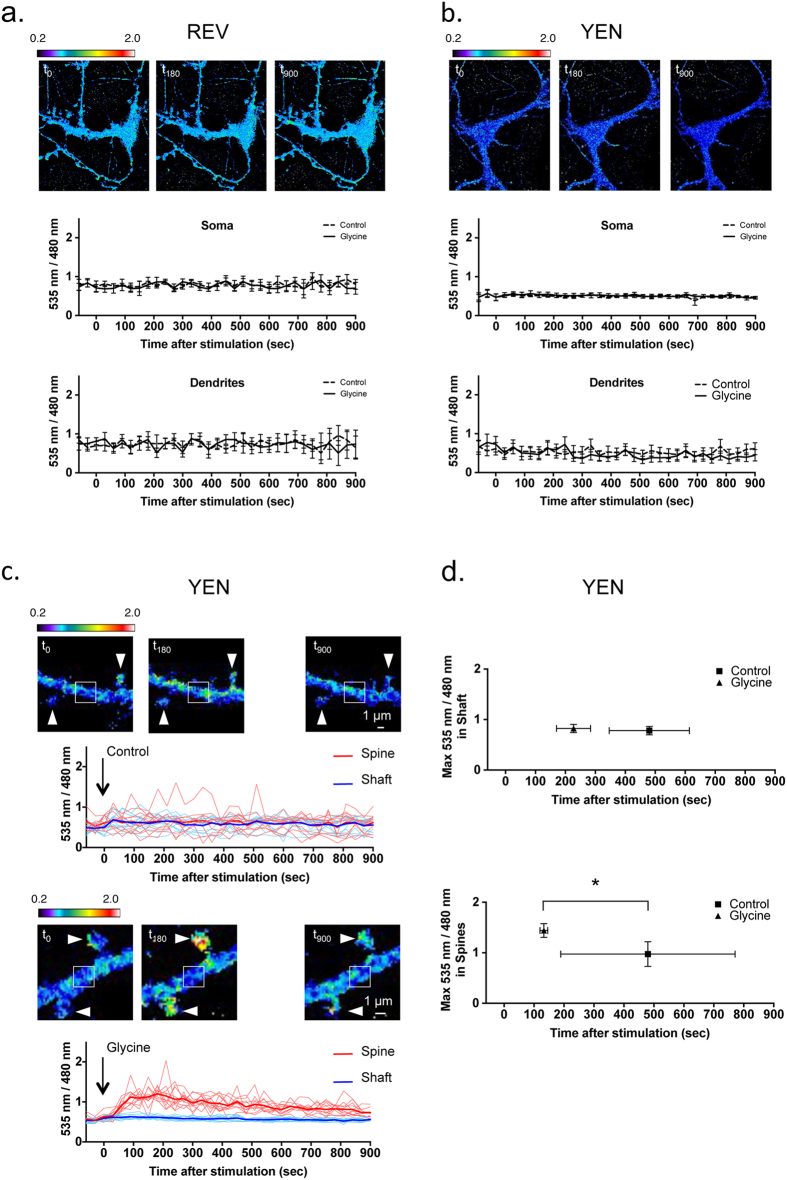
The Nluc-containing reporter is able to detect subtle ERK-activity modulations in the dendritic spines of neurons. **(a,b)** The intensities of the 535 nm/480 nm ratio over time, recorded in the soma or dendrites of REV- (**a**) or YEN- (**b**) transfected neurons, stimulated or not (control) with Glycine. One representative image illustrates the 535 nm/480 nm ratio at the indicated times. Each point of the graphs represents the mean ± SEM for 3 to 6 neurons and 7 regions per neuron, for each time point. **(c)** The 535 nm/480 nm ratio of individual spine (arrowhead) and shaft (scare) areas were measured over time. Bold traces represent lines of best fit from 12 traces. One representative image illustrates the 535 nm/480 nm ratio at the indicated times. **(d)** The intensity (Y axis) and time position (X axis) of the maximal 535 nm/480 nm ratios measured in 12 shaft areas (upper panel) and 12 spines (bottom panel) in the absence and presence of glycine. Each plot is the mean ± SEM of the time positions and mean ± SEM of the peak intensities. Mann & Whitney statistical analysis: *p < 0.05. Glycine stimulation induced a significant increase of the maximal BRET intensity in spines, compared to non-stimulated neurons.

We then took advantage of YEN by investigating whether there existed any ERK modulations that would have previously been beyond the detection limits of REV^15^. Specifically, we assessed ERK activity in cultured hippocampal neurons following synaptic NMDA receptor activation by application of the co-agonist glycine (a protocol validated to induce chemical long-term potentiation^16^). Following glycine induction, neither REV- nor YEN- BRET signals (measured on the soma or dendritic processes over time) were significantly different from the non-stimulated condition (Fig. 6a,b). However, the high intensity of the Nluc light emission reduced the YEN BRET image acquisition time (2 s versus 10 s for REV), reducing both protein movements and ERK activity changes within this short time window and markedly improving the subcellular refinement of ERK activity. Since the properties of Nluc in the YEN probe significantly improved the sensitivity, temporal and spatial resolution and duration of BRET signal stability, we were able to refine the YEN BRET analysis to compare dendritic shafts versus dendritic spines. Under control conditions, YEN displayed a stable 535 nm/480 nm ratio over time, both in dendritic shaft and spine areas (Fig. 6c, upper panel). However, analysis of the 535 nm/480 nm ratio intensities between individual spines highlighted a stochastic ERK activity, probably attributable to differences in the synaptic electrical and biochemical activity between spines. Interestingly, glycine application induced a marked and homogenous increase in 535 nm/480 nm ratio intensities in dendritic spines, with as little as a 60 s incubation (Fig. 6c, bottom panel). The maximal 535 nm/480 nm ratio in spines rose by 132.85 ± 12.85 s after glycine application and was significantly higher (1.44 ± 0.13) than the maximal 535 nm/480 nm ratio measured in spines in the absence of stimulation (0.97 ± 0.24, Fig. 6c). No significant increase was observed in dendritic shafts (Fig. 6c,d). Hence, YEN enabled us to report a subtle, spine-confined increase in ERK activity following endogenous synaptic NMDA stimulation.

## Discussion

Using a bright luciferase, we have characterized a faster and higher resolution single-cell BRET imaging technique. The time resolution and stability of BRET signals over time were substantially improved. The spatio-temporal noise was also reduced and the dynamic window was enlarged, increasing the sensitivity and reliability of the BRET signal.

The brightness of Nluc enabled us to decrease the minimal acquisition time to 200 ms per BRET image, outperforming the possibilities offered by improvements to FRET imaging^14^,^17^. While the mobility of proteins was reduced due to shorter acquisition times, the spatial resolution of BRET images was also substantially enhanced. Increased Nluc brightness also meant that a lower number of donor entities were required to detect a reliable BRET signal. This will be useful in recording the interactions of a small number of proteins that are confined to narrow subcellular compartments and will also be essential for cell-population BRET to assess protein-protein interactions of weakly expressed proteins. Future developments of BRET imaging may include a beam splitter placed in front of the camera to separate the light bellow and upper 500 nm, to collect simultaneously on 2 different areas of the camera the light coming from the donor and the light coming from the acceptor. This configuration would halve the acquisition time and further refine the resolution of BRET in space and time, without any loss in sensitivity since even more light would be collected than using the actual filters (see Fig. S1c). Alternatively, filter optimization centered on an Nluc peak of luminescence at 440 nm would also further enhance the detection of fewer donor units.

The left-shifted peak of Nluc enhanced the spectral separation of its emission from the Venus acceptor emission (Fig. S1a,b), confirming previous reports^7^,^18^. Consequently, the minimized overflow of the energy donor emission in the acceptor detection channel reduced the basal 535 nm/480 nm ratio of Nluc-tagged proteins. We also reported a higher 535 nm/480 nm ratio within the Nluc-Venus fusion compared to Rluc8-Venus. The efficiency of BRET primarily relies on the overlap between donor emission and acceptor excitation spectra. It is interesting to note that despite its left-shifted spectrum the intense Nluc emission efficiently excited Venus. However, the distance and relative orientation between donor and acceptor entities has also been shown to be a key factor determining BRET efficiency^19^,^20^. Thus, the increase in the 535 nm/480 nm ratio might essentially report a favorable orientation between Nluc and Venus in the fusion, which could vary unpredictably between constructions. In line with this observation, the 535 nm/480 nm ratio of the YEN ERK reporter was only slightly increased compared to REV. However, in both experiments the low basal 535 nm/480 nm ratio and efficient energy transfer initiated by Nluc substantially enlarged the dynamic range of the BRET signal. The resulting increased dynamic window together with a 2-fold reduced temporal noise in Nluc constructs considerably improved the sensitivity of the assay. Furthermore, the longer-lasting intensive brightness of Nluc increased the stability of the BRET signal over time, allowing protein remodeling to be studied for more than 1 hour.

We engineered YEN in order to illustrate the benefits of Nluc for unravelling complex intracellular processes within single living cells. In neurons, dendritic spines are small, labile protrusions hosting intense and highly dynamic electrical and biochemical signals which make the acquisition of BRET signals particularly challenging. However, the decrease in acquisition time with our YEN sensor allowed us to reliably monitor BRET signals at the spine level, revealing subtle, transient and localized ERK activity modulations induced by the activation of endogenous synaptic NMDA receptors. ERK activation is a point of convergence for many distinct signaling pathways involved in the regulation of various physiological processes ranging from cell proliferation and differentiation to cell survival and apoptosis^21^. Thus, YEN will help to unravel the complexity of ERK signaling in these physiological processes, including neuronal plasticity.

To conclude, the demonstrated benefits of using Nluc as the energy donor for single-cell BRET imaging further justify the recent enthusiasm for the use of Nluc in improving luminescent assays such as receptor-ligand binding assays^18^, protein aggregation^22^ or *in vivo* optical imaging^23^. The use of Nluc as an energy donor in BRET imaging should provide a better description of intracellular events such as protein-protein interactions and the spatio-temporal activation patterns of signaling pathways. The technology described here for the improved imaging of very dim signals, should also enable a multitude of new applications for imaging without fluorescent excitation, thereby avoiding the drawbacks of photobleaching, autofluorescence, phototoxicity and undesirable stimulation of photobiological processes. In particular, BRET imaging provides great potential for studies investigating the deep penetration of animal tissues.

## Methods

### Plasmids

The complete DNA coding sequence of Nanoluciferase (Nluc) was amplified by PCR with or without its STOP codon and ligated into the empty mammalian expression vector pcDNA3 between the BamHI/HindIII restriction sites to generate the pcDNA3-Nluc plasmid. The coding sequence of Nluc without the STOP codon was fused in-frame to the N-terminus of the coding sequence of Venus via a 6-amino-acid linker (GSPGTG) between the HindIII/BamHI restriction sites of the pcDNA3.1-Venus to generate the fusion coding plasmid pcDNA3.1-Nluc-Venus. The fusion plasmid pcDNA3.1-Rluc8-Venus was generated by replacing the sequence coding for Nluc with that coding for Rluc8 between the HindIII/BamHI sites in the pcDNA3.1-Nluc-Venus plasmid. The REV coding plasmid has been previously described^15^. The plasmid coding for YEN contains a cytoplasmic Extracellular signal-regulated Kinase Activity Reporter (EKAR) boxed between BRET-compatible entities (Ypet and Nluc), pEevee-Ypet-EKARcyto-Nluc. It was designed from the optimized backbone structure of a FRET biosensor with the 116-amino-acid (SAGG)_29_-repeats linker (pEKAREV(3560)NES pEevee^24^). The ECFP-coding sequence, removed by NotI/XbaI digestion, was replaced by the Nluc-coding sequence, amplified by PCR (from pNanoLuc3.2 NFKB RE) and inserted into the NotI/XbaI sites by an Infusion reaction (Clontech), as recommended by the manufacturer. This cloning process preserved the C-terminus Nuclear Export Signal (NES), maintaining the reporter protein in the cytosol. The YEN sensor has been optimized in three major areas compared to the previous REV sensor. It contains: (i) a longer flexible linker, (ii) YPet instead of Venus, and (iii) Nluc instead of Rluc8.

### Reagents

Glycine (200 μM) and strychnine (1 μM) were purchased from Sigma-Aldrich, St Quentin Fallavier, France. Coelenterazine H and Furimazine were obtained from Promega, Charbonnières-les-bains, France.

### Cell culture and transfection

Human embryonic kidney 293 cells (HEK293 T) were cultured and transfected as previously described^25^. Distinct pools of cells were transfected with either the plasmid coding for the donor-acceptor fusion (pcDNA3.1-Nluc-Venus or pcDNA3.1-Rluc8-Venus, 500 ng) or plasmids coding for the donor entity alone (pcDNA3-Nluc or pcDNA3.1-Rluc8, 500 ng) together with pDsRed-N1 (500 ng) as a transfection reporter. A suitable amount of the non-coding plasmid pcDNA3 was also added to reach a total amount of 5 μg of DNA per 100 mm diameter dish (3,000,000 cells).

### Culture of primary hippocampal neurons

Hippocampal neuronal primary cultures were prepared from embryonic day 17.5 mice as previously described^15^. Briefly, brain hippocampi were digested with trypsin and hippocampal cells were seeded in neurobasal medium (NB) (Gibco, Invitrogen, Cergy Pontoise, France) supplemented with 2% B-27 (Gibco), 4 mM glutamax (Gibco), 25 μM glutamic acid (Gibco), 100 U/ml Penicillin, 100 μg/ml Streptomycin and 10% Fetal Bovine Serum (FBS), in 35 mm diameter glass bottom culture dishes. After 3 days in culture (DIV3), the culture medium was supplemented with 5 μM cytosine β-d-arabinofuranoside hydrochloride (Sigma-Aldrich) for 12 hours. Then, 75% of the medium was replaced by NB supplemented with B-27, glutamax and antibiotics. Neurons were then transfected with 100 ng of the pEevee-Nluc-EKARcyto-Ypet plasmid and 1.9 μg of the non-coding plasmid pcDNA3 using Lipofectamine 2000 (Invitrogen, Cergy Pontoise, France), according to the manufacturer’s standard protocol at DIV10, and were recorded between DIV11 and DIV12.

For BRET recordings, neurons were incubated in the following external medium: 140 mM NaCl, 2 mM CaCl_2_, 3 mM KCl, 10 mM Hepes, 10 mM d-glucose, 0.01 mM glycine, pH 7.4, with an osmolarity of 330 mOsm. To stimulate synaptic NMDA receptors, this medium was complemented with saturating levels (200 μM) of the co-agonist glycine and 1 μM strychnine^16^.

Ethics statement: Primary cell cultures were performed in accordance with relevant guidelines and regulations. The experimental protocol was approved by the institutional committee « *Comité National de Réflexion Ethique sur l’Expérimentation Animale »*; protocol # CEEA-LR-12025

### Single-cell BRET imaging

Single-cell BRET imaging experiments were performed as previously described^2^,^3^. Briefly, distinct pools of cells were transfected with plasmids coding for the donor-Venus fusion or plasmids coding for the donor alone and DsRed as a transfection reporter. 24 hours after transfection, the two populations of transfected cells expressing the donor-Venus fusion or the donor alone were pooled and cultured for an additional 24 hours in glass-bottom culture dishes (MatTek Corporation, Ashland, MA, USA). Thus, we were able to record the 535 nm/480 nm ratio signals originating from cells expressing Nluc-Venus or Rluc8-Venus under the same conditions as the basal 535 nm/480 nm ratio, which resulted from Nluc- or Rluc8-transfected cells in the same microscopic field (Fig. 1). All images were obtained using a bioluminescence-dedicated inverted fluorescence microscope (Axiovert 200M; Carl Zeiss) with a Plan Apochromat 63×/1.40 oil M27 objective at room temperature and collected with an evolve camera (Photometrics) equipped with an EMCCD detector, back-illuminated, On-chip Multiplication Gain. Sequential acquisitions of light emission at 480 nm and 535 nm wavelengths (Em480 and Em535) were performed at 5 MHz, gain 3950, binning 1, with emission filters D480/60 nm (No. 61274, Chroma) and HQ535/50 nm (No. 63944, Chroma) respectively, for the indicated acquisition times. Coelenterazine H (20 μM) or Furimazine (50 μM) were applied 5 min before acquisition using MetaMorph software (Molecular Devices), except in the case of kinetic experiments. The average intensities or background signals of Em480, Em535 and the 535 nm/480 nm ratios were recorded in a square area of 21 × 21 pixels drawn on a cell of interest or in an adjacent cell-free region, respectively. The signal/background ratio corresponded to the previous average intensity signal over the mean background signal. The background was subtracted from the Em480 and Em535 raw images. The 535 nm/480 nm ratio images were generated by pixel-by-pixel division of the absolute light intensities at 535 nm and 480 nm. In the ratio images, absolute numerical 535 nm/480 nm ratios were translated and visualized with a continuous 256-pseudocolor look-up table comprised between the minimum and maximum values indicated on the corresponding scale.

For kinetics experiments, the 535 nm/480 nm ratio was measured every minute, starting at the addition of the luciferase substrate. To calculate the temporal noise, we measured the mean standard deviation of the 535 nm/480 nm ratio from 5 to 20 min after substrate addition and expressed it as a percentage of the average 535 nm/480 nm ratio. The spatial noise was reported by taking the standard deviation among pixels within a square area of 21 × 21 pixels and expressing it as a percentage of the mean intensity of the 535 nm/480 nm ratio in this area.

### Luminescence and BRET Spectra

48 hours after transfection, cells were suspended in 0.1% PBS glucose at room temperature and distributed in 96-well microplates at a density of 50,000 cells/well. Immediately before measurement, Coelenterazine H or Furimazine were added at a final concentration of 5 μM and 20 μM respectively. Spectral analysis (Fig. S1) was performed using the spectral scanning function of a Flexstation plate reader (Molecular Device, 20 nm bandwidth, 5 nm step size, integration time 0.5 s).

### Cell population BRET measurements

BRET measurements in cell populations (Fig. S2) were performed as previously described^25^ using the Mithras LB 940 (Berthold Technologies). 48 hours after transfection, cells were suspended in 0.1% PBS glucose at room temperature. Cells were then distributed in 96-well micro-plates at a density of 10,000 cells/well. Before the BRET experiment, we measured the light emitted at 530 nm upon light excitation at 485 nm, indicative of the amount of Venus-tagged proteins. BRET, or the 535 nm/485 nm ratio, was assessed by calculating the ratio of the light emitted by the acceptor entity (510–550 nm band-pass filter, Em535) to the light emitted by the donor (460–500 nm band-pass filter, Em480) after the addition of 5 μM Coelenterazine H or 20 μM Furimazine. For time course experiments, BRET was measured every 2 min for 1 hour.

### Data computation and statistical analysis

To predict the 535 nm/480 nm ratios from luminescence spectra, the area under curve within the theoretical limits of the 450–510 nm and the 510–560 nm band-pass filters used in BRET imaging were calculated with the GraphPad Prism 6 software (GraphPad Software Inc.) to estimate the theoretical donor and acceptor emissions respectively.

Statistical analysis was carried out with the GraphPad Prism 6 software, using the non-parametric Mann & Whitney test for two independent samples, the Kruskal & Wallis test for more than two independent samples and the Friedman test for paired samples with a P risk threshold of 5%.

## Additional Information

**How to cite this article**: Goyet, E. *et al*. Fast and high resolution single-cell BRET imaging. *Sci. Rep.* **6**, 28231; doi: 10.1038/srep28231 (2016).

## Supplementary Material

Supplementary Information
