# Supplementary material for: Fast and high resolution single-cell BRET imaging
Source: Sci Rep. 2016 Jun 15;6:28231. doi: 10.1038/srep28231 (PMC4908377; doi:10.1038/srep28231)
Supplement: Supplementary Information [file srep28231-s1.pdf]

Fast and high resolution single-cell BRET imaging

Elise Goyet [1,2,3], Nathalie Bouquier [1,2,3], Vincent Ollendorff [4] and Julie Perroy [1,2,3]

[1] CNRS, UMR-5203, Institut de Génomique Fonctionnelle, Montpellier, F-34094, France

[2] INSERM, U1191, Montpellier, F-34094, France

[3] Universités de Montpellier, UMR-5203, Montpellier, F-34094, France

[4] INRA, UMR866 Dynamique Musculaire et Mébabolisme, Université Montpellier, 34060 Montpellier, France

Correspondence should be addressed to:

Julie Perroy
IGF, CNRS, Montpellier
Julie.perroy@igf.cnrs.fr

Short title: *Improving BRET imaging*

Key words: Bioluminescence Resonance Energy Transfer (BRET) Imaging – Nanoluciferase – protein-protein interaction – protein conformational changes - Extracellular-signal-Regulated Kinase (ERK) – cell signaling – neurons.

SUPPLEMENTAL DATA

Spectral properties of Nluc

Donor emission spectra

As a prelude to BRET imaging experiments, we first explored the spectral properties of Nluc. We recorded the light emission spectra of Rluc8- or Nluc-transfected HEK293 cells in the presence of their enzymatic substrate (Coelenterazine H or Furimazine, respectively). For equal amounts of transfected DNA, Nluc-transfected cells displayed a 3-fold higher peak of luminescence intensity than Rluc8-transfected cells (Fig. S1a). The Nluc emission peak was left-shifted by 40 nm compared to the Rluc8 emission peak (446.25 ± 1.25 nm versus 487.50 ± 1.44 nm), as seen when both spectra were normalized to their own emission peaks (Fig. S1b). Upon drawing the band-pass of filters to be used in our microscope to perform BRET imaging we could already predict the benefits of Nluc. By measuring the area under the curve within the wavelength limits of the donor (480 ± 30 nm) and acceptor (535 ± 25 nm) emission filters, we calculated the proportion of energy donor output that would overflow into the energy acceptor detection filter. Nluc emission displayed a 2-fold smaller basal 535 nm /480 nm ratio than Rluc8 (0.2093 ± 0.0049 for Nluc versus 0.5031 ± 0.0177 for Rluc8, Fig. S1e). Hence, the left-shifted peak of Nluc enabled a better spectral separation of the donor emission from the Venus acceptor emission. This would be expected to improve the dynamic range of BRET signals with the Nluc/Venus couple compared to the Rluc8/Venus couple.

BRET emission spectra

To assess the performances of Rluc8 and Nluc in exciting the BRET-compatible acceptor, Venus, we used genetically-fused donor and acceptor entities, Rluc8-Venus and Nluc-Venus, as BRET-positive fusions. We measured the emission spectra of light induced by

the catalytic oxidation of Coelenterazine H or Furimazine, in HEK cells expressing Rluc8-Venus or Nluc-Venus, respectively. For the same Venus fluorescence intensity i.e. the same amount of expressed fusion units, the Nluc luminescence output was substantially higher than that of Rluc8 (Fig. S1c).

The peak of light emitted by Venus, resulting from energy transfer within the fusion, was higher when Nluc was used as donor entity compared to Rluc8 (Fig. S1d). The BRET (535 nm / 480 nm ratio) estimated from the calculation of the area under curve within the limits of acceptor and donor filters was significantly higher for Nluc/Venus than for Rluc8/Venus (1.2030 ± 0.0525 versus 0.7838 ± 0.0169, Fig. S1e). When subtracting the basal 535 nm / 480 nm ratio, the net 535 nm / 480 nm ratio, i.e. the signal originating from only the energy transfer, was higher with Nluc, suggesting a more efficient energy transfer within the Nluc-Venus fusion compared to Rluc8-Venus (0.99 ± 0.05 versus 0.28 ± 0.02, Fig. S1f).

The better spectral separation resulting from the left-shifted spectrum of Nluc predicted an increased dynamic range. Moreover, because of its substantially greater brightness, the detection of Nluc luminescence was expected to require a reduced acquisition time with the same number of light units. Alternatively, it would allow working with low expression levels of donor-tagged entities which would be useful at the single-cell level.

Figure S1

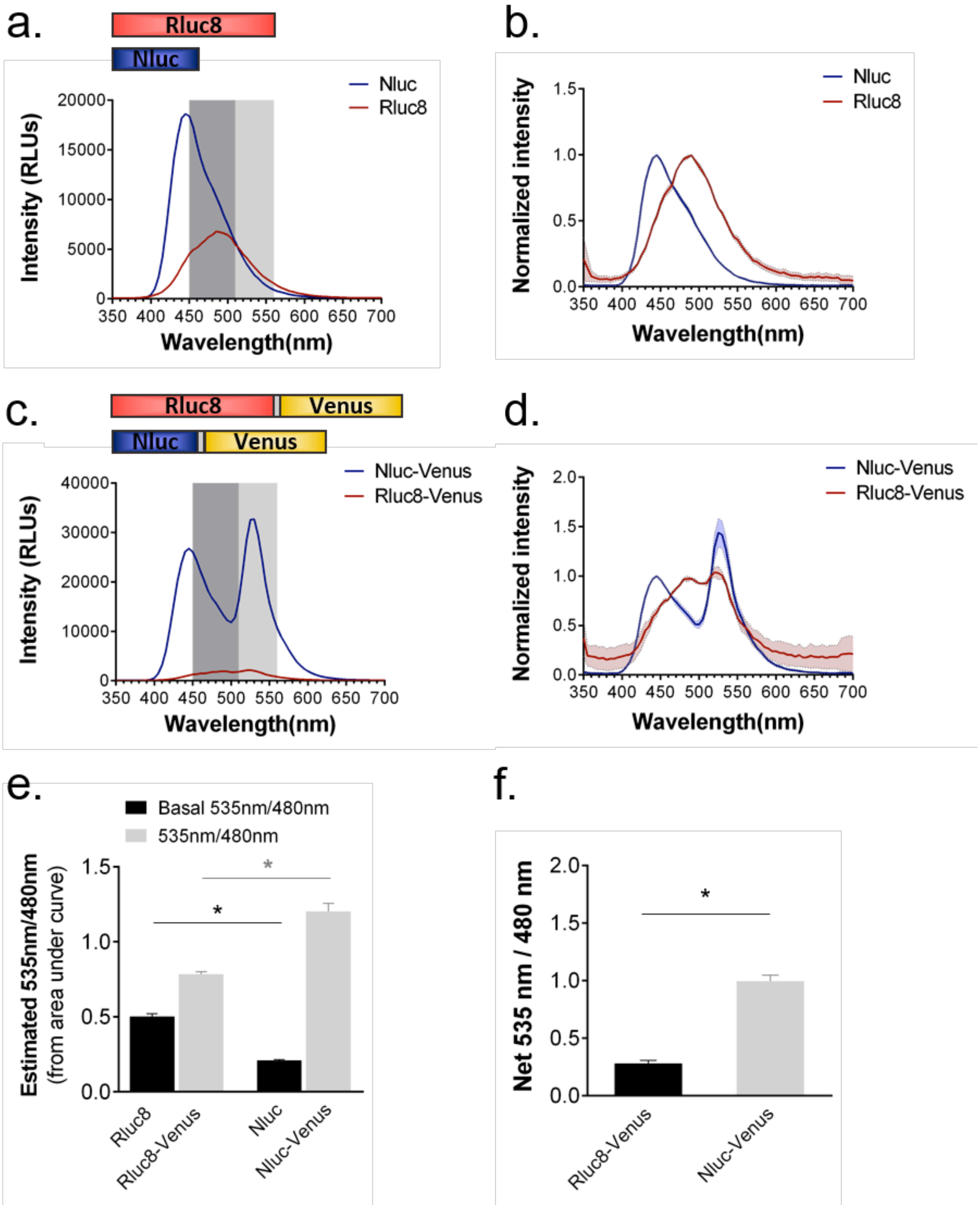

a.
Rluc8
Nluc
Nluc
Rluc8
Intensity (RLUs)
20000
15000
10000
5000
0
350 400 450 500 550 600 650 700
Wavelength(nm)
b.
Nluc
Rluc8
Normalized intensity
1.5
1.0
0.5
0.0
350 400 450 500 550 600 650 700
Wavelength(nm)
c.
Rluc8
Venus
Nluc
Venus
Nluc-Venus
Rluc8-Venus
Intensity (RLUs)
40000
30000
20000
10000
0
350 400 450 500 550 600 650 700
Wavelength(nm)
d.
Nluc-Venus
Rluc8-Venus
Normalized intensity
2.0
1.5
1.0
0.5
0.0
350 400 450 500 550 600 650 700
Wavelength(nm)
e.
Basal 535nm/480nm
535nm/480nm
Estimated 535nm/480nm
(from area under curve)
1.5
1.0
0.5
0.0
*
*
Rluc8
Rluc8-Venus
Nluc
Nluc-Venus
f.
Net 535 nm / 480 nm
2.0
1.5
1.0
0.5
0.0
*
Rluc8-Venus
Nluc-Venus

Fig. S1: The high left-shifted peak of Nluc improves the resolution between donor and Venus acceptor emission spectra.

(A – D) Luminescence spectra of Nluc and Rluc8 (A and B) or Nluc-Venus and Rluc8-Venus (C and D) induced by the catalytic oxidation of their respective enzymatic substrates, Furimazine and Coelenterazine H. Light intensities are represented as absolute values recorded from one representative experiment (A and C) or as the mean ± SEM of 4 independent experiments normalized to the peak of donor emission (B and D). Note that similar amounts of DNA coding for Nluc and Rluc8 were transfected into cells in A and B, while Venus fluorescence indicated the same levels of protein expression in C and D. The Nluc emission peak was left-shifted by 40 nm compared to that of Rluc8, improving the spectral resolution with Venus. RLUs = relative light units. **(E)** 535 nm / 480 nm ratios of Nluc, Rluc8, Nluc-Venus and Rluc8-Venus estimated from calculating the area under the curve. Theoretical wavelength filters used to calculate donor and acceptor emissions are drawn in dark and light grey respectively in (A) and (C). While the basal 535 nm / 480 nm ratio was decreased for Nluc compared to Rluc8, the 535 nm / 480 nm ratio was increased for Nluc-Venus compared to Rluc8-Venus. **(F)** Net 535 nm / 480 nm ratios of Rluc8-Venus and Nluc-Venus calculated by subtracting the basal 535 nm / 480 nm ratio of Rluc8 and Nluc from the 535 nm / 480 nm ratio of Rluc8-Venus and Nluc-Venus respectively. Consequently, the calculated net 535 nm / 480 nm ratio was significantly higher for Nluc-Venus than Rluc8-Venus. Mann & Whitney statistical analysis: *, $p<0.05$.

Nluc in cell-population BRET

We further studied the possible benefits of using Nluc in a cell-population BRET assay, by measuring the Em480, Em535 and 535 nm / 480 nm ratio in cell populations with a fluorimeter. As highlighted by the emission spectra of Rluc8 and Nluc (Fig. S1), for equivalent levels of fusion unit expression, the Em480 intensity in Nluc-Venus-transfected cells was substantially increased compared to Rluc8-Venus in the presence of their specific substrates, Furimazine and Coelenterazine H respectively (Fig. S2a). The Em480 intensity reached a maximum within 2 minutes after substrate addition, and the subsequent decrease in light was slower for Nluc than Rluc8 (Fig. S2b). The Em480 signal of Rluc8 decreased to 50 % of its maximum after 25 min of Coelenterazine H incubation while the Em480 signal of Nluc remained higher than 50 % of its maximum for over 1 hour after Furimazine addition (Fig. S2b). The Em535 signals displayed the same kinetics as Em480 (data not shown). Consequently, the 535 nm / 480 nm ratio signal in the cell population was easily detectable for more than 1 hour after incubation with the relevant substrate for both luciferases (Fig. S2c). It is also interesting to note that Nluc can also catalyze the oxidation of Coelenterazine H, giving rise to an intense light emission just after substrate addition (equivalent to the intensity of light reached by Nluc oxidation of Furimazine) but with a more rapid decrease over time (even faster than the decrease in light emitted by Coelenterazine H oxidation by Rluc8). These first results show that although the light intensity emitted by Nluc is higher, both donors can be efficiently used to accurately report BRET signals for up to 1 hour at the cell population level. However, when comparing the acceptor-donor fusion to when the donor was expressed alone, the use of Nluc significantly enlarged the dynamic window in cell-population assays compared to Rluc8 (reported by the net 535 nm / 480 nm ratios, Fig. S2e). Finally, in cell populations, the noise of the 535 nm / 480 nm ratio (measured between 5 and 20 min) was not significantly different when using Nluc or Rluc8 (Nluc-Venus: 0.80 ± 0.19 %

versus Rluc8-Venus: 0.91 ± 0.16 % for the 535nm / 480nm ratio, Fig. S2f). Thus the main benefit of using Nluc in cell-population BRET assays is the enlargement of the dynamic window that will allow subtle variations of BRET to be reported. Nluc might also be preferred when studying proteins with excessively low levels of expression.

Figure S2

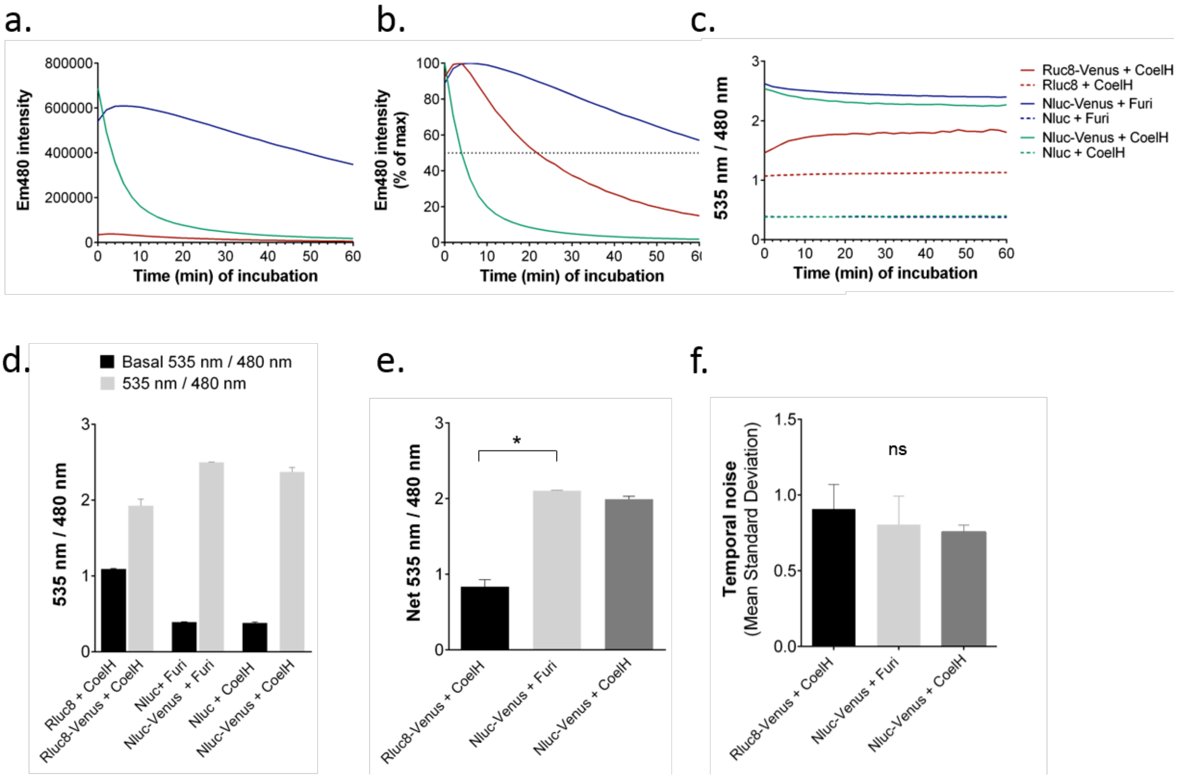
a.
b.
c.
d.
e.
f.
Em480 intensity
Time (min) of incubation
Em480 intensity (% of max)
535 nm / 480 nm
Ruc8-Venus + CoelH
Rluc8 + CoelH
Nluc-Venus + Furi
Nluc + Furi
Nluc-Venus + CoelH
Nluc + CoelH
Basal 535 nm / 480 nm
535 nm / 480 nm
Rluc8 + CoelH
Rluc8-Venus + CoelH
Nluc+ Furi
Nluc-Venus + Furi
Nluc + CoelH
Nluc-Venus + CoelH
Net 535 nm / 480 nm
*
Temporal noise (Mean Standard Deviation)
ns

Fig. S2: Benefits of Nluc versus Rluc8 as an energy donor in cell-population BRET.

(A-C) Signal intensities for the equivalent amount of YFP Fluorescence under each condition. **(A)** Em480 signal. **(B)** Em480 signal expressed as a percentage of the maximum intensity. **(C)** The 535 nm / 480 nm ratio originating from the oxidation of Furimazine and Coelenterazine H by Nluc and Rluc8 over time. Graphs are representative of 3 independent experiments. **(D)** Bar graph of the average 535 nm / 480 nm ratio intensities of Rluc8, Rluc8-Venus, Nluc and Nluc8-Venus between 5 and 20 min of Coelenterazine H or Furimazine incubation. Note that Nluc decreased the basal 535 nm / 480 nm ratio and triggered a more efficient energy transfer with Venus for both substrates (Coelenterazine H or Furimazine) compared to Rluc8. **(F)** Net 535 nm / 480 nm ratios of Nluc-Venus and Rluc8-Venus between 5 and 20 min of Coelenterazine H or Furimazine calculated by subtracting the basal 535 nm / 480 nm ratio of Rluc8 and Nluc from the 535 nm / 480 nm ratio of Rluc8-Venus and Nluc-Venus respectively. As a result, the dynamic window for 535 nm / 480 nm ratio measurements was significantly improved. **(E)** Mean standard deviation of the 535 nm / 480 nm ratio between 5 and 20 min after substrate addition, expressed as a percentage of the mean ratio intensity. Bars represent the mean ± SEM obtained from 3 independent experiments. Kruskal-Wallis statistical analysis: *, $p<0.05$.
